# Influenza hospitalizations in Australian children 2010–2019: The impact of medical comorbidities on outcomes, vaccine coverage, and effectiveness

**DOI:** 10.1111/irv.12939

**Published:** 2021-11-16

**Authors:** Daniel A. Norman, Allen C. Cheng, Kristine K. Macartney, Hannah C. Moore, Margie Danchin, Holly Seale, Jocelynne McRae, Julia E. Clark, Helen S. Marshall, Jim Buttery, Joshua R. Francis, Nigel W. Crawford, Christopher C. Blyth

**Affiliations:** ^1^ Wesfarmers Centre of Vaccines and Infectious Diseases, Telethon Kids Institute University of Western Australia Nedlands Western Australia Australia; ^2^ School of Medicine University of Western Australia Crawley Western Australia Australia; ^3^ Infection Prevention and Healthcare Epidemiology Unit Alfred Health Melbourne Victoria Australia; ^4^ School of Public Health and Preventive Medicine Monash University Melbourne Victoria Australia; ^5^ National Centre for Immunisation Research and Surveillance of Vaccine Preventable Diseases The Children's Hospital at Westmead Westmead New South Wales Australia; ^6^ Department of Infectious Diseases and Microbiology The Children's Hospital at Westmead Westmead New South Wales Australia; ^7^ Discipline of Child and Adolescent Health, Faculty of Medicine University of Sydney Sydney New South Wales Australia; ^8^ Department of Paediatrics University of Melbourne Parkville Victoria Australia; ^9^ Vaccine Hesitancy Murdoch Children's Research Institute Parkville Victoria Australia; ^10^ Department of General Medicine The Royal Children's Hospital Parkville Victoria Australia; ^11^ School of Population Health University of New South Wales Randwick New South Wales Australia; ^12^ Infection Management and Prevention Service Queensland Children's Hospital South Brisbane Queensland Australia; ^13^ Adelaide Medical School The University of Adelaide Adelaide South Australia Australia; ^14^ Robinson Research Institute The University of Adelaide Adelaide South Australia Australia; ^15^ The Vaccinology and Immunology Research Trials Unit Women's and Children's Health Network Adelaide South Australia Australia; ^16^ Department of Infection and Immunity Monash Children's Hospital, Monash Health Clayton Victoria Australia; ^17^ Monash Centre of Health Care Research and Implementation, Departments of Paediatrics Monash University Melbourne Victoria Australia; ^18^ Royal Darwin Hospital Top End Health Service Darwin Northern Territory Australia; ^19^ Menzies School of Health Research Charles Darwin University Darwin Northern Territory Australia; ^20^ SAFEVIC Murdoch Children's Research Institute Parkville Victoria Australia; ^21^ Department of Infectious Disease Perth Children's Hospital Nedlands Western Australia Australia; ^22^ PathWest Laboratory Medicine QEII Medical Centre Nedlands Western Australia Australia

**Keywords:** comorbidities, hospitalizations, pediatric influenza, vaccination

## Abstract

**Background:**

Children with comorbidities are at greater risk of severe influenza outcomes compared with healthy children. In Australia, influenza vaccination was funded for those with comorbidities from 2010 and all children aged <5 years from 2018. Influenza vaccine coverage remains inadequate in children with and without comorbidities.

**Methods:**

Children ≤16 years admitted with acute respiratory illness and tested for influenza at sentinel hospitals were evaluated (2010–2019). Multivariable regression was used to identify predictors of severe outcomes. Vaccine effectiveness was estimated using the modified incidence density test‐negative design.

**Results:**

Overall, 6057 influenza‐confirmed hospitalized cases and 3974 test‐negative controls were included. Influenza A was the predominant type (68.7%). Comorbidities were present in 40.8% of cases. Children with comorbidities were at increased odds of ICU admission, respiratory support, longer hospitalizations, and mortality. Specific comorbidities including neurological and cardiac conditions increasingly predisposed children to severe outcomes. Influenza vaccine coverage in influenza negative children with and without comorbidities was low (33.5% and 17.9%, respectively). Coverage improved following introduction of universal influenza vaccine programs for children <5 years. Similar vaccine effectiveness was demonstrated in children with (55% [95% confidence interval (CI): 45; 63%]) and without comorbidities (57% [(95%CI: 44; 67%]).

**Conclusions:**

Comorbidities were present in 40.8% of influenza‐confirmed admissions and were associated with more severe outcomes. Children with comorbidities were more likely experience severe influenza with ICU admission, mechanical ventilation, and in‐hospital morality. Despite demonstrated vaccine effectiveness in those with and without comorbidities, vaccine coverage was suboptimal. Interventions to increase vaccination are expected to reduce severe influenza outcomes.

**Abbreviations**
aOR: Adjusted odds ratio
BiPAP: Bilevel positive airway pressure
CPAP: Continuous positive airway pressure
ECMO: Extra‐corporeal membrane oxygenation
FluCAN: Influenza Complications Alert Network
ICU: Intensive care unit
IQR: Interquartile range
NIP: National Immunization Program
PAEDS: Paediatric Active Enhanced Disease Surveillance network
VE: Vaccine effectiveness

## 1 BACKGROUND

Seasonal influenza is a leading cause of pediatric morbidity and mortality globally.^1^ Young children have the highest risk of influenza‐associated hospitalization.^2^ Children with medical comorbidities including neurological, cardiac, metabolic, and hematological conditions are known to be at greater risk for severe influenza disease compared with otherwise healthy children of similar ages.^3^

Seasonal influenza hospitalizations have been captured through the *InFluenza Complications Alert Network* (FluCAN) in Australia since 2009.^4^ Two tertiary pediatric hospitals from the *Paediatric Active Enhanced Diseases Surveillance Network* (PAEDS) contributed data from 2011^5^, ^6^ expanding in 2017 with recruitment from pediatric tertiary hospitals in other states.^7^

Quadrivalent inactivated influenza vaccination is recommend for all Australian children aged ≥6 months.^8^ Live‐attenuated influenza vaccines are not available in Australia. Influenza vaccination was funded in 2010 for children aged ≥6 months old with medical comorbidities through the National Immunization Program (NIP) and Aboriginal and/or Torres Strait Islander children (herein respectfully referred to as Aboriginal) aged 6 months to <5 years in 2015.^8^ Western Australia has provided state‐based funding of influenza vaccination for children aged 6 months to <5 years old since 2008.^9^ All other Australian states and the Australian Capital Territory funded influenza vaccination for all children aged 6 months to <5 years old from 2018. Influenza vaccination for this age group was nationally funded through the NIP in 2020.^8^, ^10^

In this study, we describe the epidemiology and clinical outcomes of seasonal influenza hospitalizations in Australian children (2010–2019), focusing on children with medical comorbidities. Vaccine effectiveness (VE) against influenza‐confirmed hospitalization was evaluated using the modified incidence density test‐negative design.^11^ The impact of comorbidities on severe influenza hospitalization outcomes, vaccine coverage, and VE in Australian children is explored. These data are critical to inform the design of programs to improve vaccine coverage in those at greatest risk.

## 2 METHODS

### 2.1 Study design

Hospitalized children with an acute respiratory illness (ARI), testing positive for influenza, were enrolled from PAEDS‐FluCAN sites (2010–2019: see supporting information Table S1). Cases were enrolled during each southern hemisphere influenza season from 2010 to 2019 (April to October, inclusive). A child was eligible for enrolment as a case if they were aged ≤16 years, hospitalized with an ARI, and tested positive for influenza type A and/or B. A hospitalized case of ARI was defined by inpatient admission of the patient excluding emergency department only care with the presence of new respiratory symptoms including shortness of breath, cough, and/or rhinorrhea with or without fever. Nosocomial influenza infections with positive influenza test result were included as hospitalized ARIs.

Cases were diagnosed using respiratory specimens tested with specific influenza assays, mostly nucleic acid testing, with a small number (<3% of total cases; all tested between 2011 and 2013) diagnosed by immunofluorescence or rapid antigen testing. Influenza A and B testing was performed on all participants; however, subtyping was not routinely performed. Patients were eligible for enrolment if admitted as inpatients to a PAEDS‐FluCAN site excluding admission to the emergency department alone. Cases were classified as a nosocomial influenza infection if their first positive influenza test was dated ≥3‐day postadmission. Contemporaneously tested hospitalized children who met enrolment criteria but tested negative for influenza A and/or B were enrolled as influenza‐negative controls.

### 2.2 Assessing risk factors and outcomes

Patient demographics, age at time of hospital admission, comorbidities, clinical history, treatments, therapies, and hospitalization outcomes were collected. Comorbidities included chronic cardiac disease, chronic respiratory disease, neurological conditions, immunosuppression, malignancies, diabetes, hepatic disease, renal disease, genetic comorbidities, inborn errors of metabolism, obesity, and long‐term aspirin therapy.^12^ Immunosuppression and malignancies were combined as a single variable (immunosuppression and/or malignancy), whereas long‐term aspirin therapy and inborn errors of metabolism were grouped (other comorbidities) due to low case numbers.

Descriptive analyses of demographics, clinical characteristics, and outcomes were performed on children testing positive for influenza with grouping by influenza strains, presence of comorbidities, and comorbidity type. Categorical data were described using proportions and compared using chi‐square (*χ* ^2^) tests. Continuous data were described using medians and interquartile ranges with comparisons performed using Mann–Whitney *U* tests. Multivariable logistic regression models were used to determine the odds of intensive care unit (ICU) admission, mechanical ventilation, and mortality adjusted by age group (6–11 months, 12–23 months, 2–4 years, and ≥5 years), comorbidity, sex, Aboriginal status, antiviral treatment, and influenza type. Negative binomial regression models used the same independent variables to evaluate the impact on length of hospitalization and length of ICU stay. Variables within these models were grouped by Australian state as well as month and year of case admission.

### 2.3 Estimating vaccine coverage and effectiveness

Vaccine coverage and effectiveness estimates were undertaken using both influenza‐positive cases and test‐negative controls. Controls were patients ≤16 years old admitted to PAEDS‐FluCAN hospitals with ARI but test‐negative for influenza. Controls were randomly chosen as the most time‐proximate influenza test‐negative subjects at each site.^6^, ^7^ Influenza vaccination status was obtained through parental reporting and confirmed through the Australian Immunization Register (AIR).^13^ An immunized child was defined by receipt of at least one dose of a licensed influenza vaccine in the same calendar year and prior to date of hospital admissions. Cases and controls aged <6 months at admission, missing vaccination status, with nosocomial infection, or multiple influenza strains were removed from vaccine coverage and VE analyses (VE analysis cohort; supporting information Figure S1).

A modified incidence density test‐negative design was used to determine VE. VE was estimated as 1 minus the adjusted odds ratio (aOR) of vaccination in influenza test‐positive cases compared with test‐negative controls.^11^ Conditional logistic regression models using influenza case status as the dependent outcome were constructed for influenza vaccination. The model was adjusted for potential confounders including age at time of admission, Aboriginal status, and comorbidities and was grouped by state, month, and year of admission. All analyses were performed using Stata 16®. Ethics approval was obtained from Monash University, Australia, with reciprocal ethics and governance approvals at each site.

## 3 RESULTS

### 3.1 Demographics and clinical outcomes

From 2010 to 2019, 6057 influenza‐confirmed hospitalizations were evaluated from 20 hospital sites (supporting information Figure S1 and supporting information Table S1). The median age was 3.6 years (interquartile range [IQR]: 1.2; 7.2), 55.7% were male, and 481 (7.9%) identified as Aboriginal (Table 1). Influenza A was detected in 68.7% of cases (*n* = 4162) and Influenza B in 30.6% (*n* = 1855); 40 cases (0.7%) were positive with more than one influenza type/subtype. Most influenza A infections were not subtyped (*n* = 2866, 68.9%); however, when subtyped, influenza A/H1N1 (*n* = 647, 15.5%) and A/H3N2 (*n* = 649, 15.6%) were seen in near equal proportions. Influenza type B cases were significantly older than influenza type A cases (median age: 5.3 vs. 3.0 years*, p* < 0.001). Nosocomial influenza infection was identified in 395 cases (6.5%).

**TABLE 1:** Hospitalized influenza‐confirmed cases: Demographics, clinical factors, comorbidities, outcomes, and treatments

Variable	Influenza type, no. (%) of children					
	A/H1N1 (*n* = 647)	A/H3N2 (*n* = 649)	A/unknown (*n* = 2866)	B (*n* = 1855)	Multiple types (*n* = 40)	Total (*n* = 6057)
**Demographics**						
Male	374 (57.8%)	344 (53.0%)	1581 (55.2%)	1050 (56.7%)	23 (57.5%)	3372 (55.7%)
Aboriginal	42 (6.5%)	78 (12.0%)	217 (7.6%)	142 (7.7%)	2 (5.0%)	481 (7.9%)
Median age at admission, years (IQR)	2.8 (1.0; 5.7)	3.2 (1.1; 7.1)	3.0 (1.1; 6.5)	5.3 (2.0; 8.5)	3.0 (0.4; 6.5)	3.6 (1.2; 7.2)
**Clinical factors**						
Current influenza vaccination	24 (3.7%)	68 (10.5%)	303 (10.6%)	209 (11.3%)	2 (5.0%)	606 (10.0%)
Nosocomial infection	58 (9.0%)	37 (5.7%)	182 (6.4%)	115 (6.2%)	3 (7.5%)	395 (6.5%)
**Comorbidities**						
Any comorbidities	267 (41.2%)	284 (43.8%)	1134 (39.6%)	766 (41.3%)	18 (45.0%)	2469 (40.8%)
Respiratory comorbidity	81 (12.5%)	95 (14.6%)	410 (14.3%)	243 (13.1%)	5 (12.5%)	834 (13.9%)
Neurological comorbidity	62 (9.6%)	79 (12.2%)	271 (9.5%)	203 (10.9%)	5 (12.5%)	620 (10.2%)
Immunosuppression and/or malignancy	63 (9.7%)	70 (10.8%)	280 (9.8%)	197 (10.6%)	4 (10.0%)	614 (10.1%)
Cardiac comorbidity	25 (3.9%)	38 (5.9%)	156 (5.4%)	109 (5.9%)	2 (5.0%)	330 (5.5%)
Genetic comorbidity	14 (2.2%)	35 (5.4%)	128 (4.5%)	91 (4.9%)	3 (7.5%)	271 (4.5%)
Renal comorbidity	17 (2.6%)	25 (3.9%)	71 (2.5%)	59 (3.2%)	1 (2.5%)	173 (2.9%)
Hepatic comorbidity	7 (1.1%)	9 (1.4%)	64 (2.2%)	42 (2.3%)	0 (0.0%)	122 (2.0%)
Diabetes	4 (0.6%)	11 (1.7%)	32 (1.1%)	29 (1.6%)	0 (0.0%)	76 (1.3%)
Obesity	3 (0.5%)	5 (0.8%)	16 (0.6%)	13 (0.7%)	0 (0.0%)	37 (0.6%)
Other comorbidities	4 (0.6%)	1 (0.2%)	23 (0.8%)	5 (0.3%)	0 (0.0%)	33 (0.5%)
**Number of distinct comorbidity types**						
0	380 (58.7%)	365 (56.2%)	1732 (60.4%)	1089 (58.7%)	22 (55.0%)	3588 (59.2%)
1	192 (29.7%)	157 (24.2%)	703 (29.7%)	480 (25.9%)	11 (27.5%)	1543 (25.5%)
2	56 (8.7%)	85 (13.1%)	281 (9.8%)	186 (10.0%)	4 (10.0%)	612 (10.1%)
3	17 (2.6%)	32 (4.9%)	99 (3.5%)	67 (3.6%)	3 (7.5%)	218 (3.6%)
4	1 (0.2%)	6 (0.9%)	38 (1.3%)	26 (1.4%)	0 (0.0%)	71 (1.2%)
≥5	1 (0.2%)	4 (0.6%)	13 (0.5%)	7 (0.4%)	0 (0.0%)	25 (0.4%)
**Treatments**						
Antiviral use	155 (24.0%)	191 (29.4%)	566 (19.8%)	377 (20.3%)	13 (32.5%)	1302 (21.5%)
Oxygen support	46 (7.1%)	56 (8.6%)	135 (4.7%)	71 (3.8%)	1 (2.5%)	309 (5.1%)
Noninvasive support (CPAP/BiPAP)	14 (2.2%)	10 (1.5%)	87 (3.0%)	50 (2.7%)	1 (2.5%)	162 (2.7%)
Mechanical ventilation	25 (3.9%)	20 (3.1%)	111 (3.9%)	52 (2.8%)	2 (5.0%)	210 (3.5%)
ECMO	1 (0.2%)	1 (0.2%)	4 (0.1%)	7 (0.4%)	1 (0.3%)	14 (0.2%)
**Hospitalization outcomes**						
Length of stay; median, days (IQR)	2 (1; 4)	2 (1; 4)	2 (1; 3)	2 (1; 3)	2 (1; 3)	2 (1; 3)
ICU admission	81 (12.5%)	65 (10.0%)	333 (11.6%)	186 (10.0%)	4 (10.0%)	669 (11.1%)
ICU length of stay: median, days (IQR)	2 (1; 6)	2 (1; 4)	2 (1; 6)	3 (1; 7)	11 (4; 33)	2 (1; 6)
Mortality	2 (0.3%)	3 (0.5%)	9 (0.3%)	11 (0.6%)	0 (0.0%)	25 (0.4%)

Abbreviations: BiPAP, bilevel positive airway pressure; CPAP, continuous positive airway pressure; ECMO, extra‐corporeal membrane oxygenation; ICU, intensive care unit; IQR, interquartile range.

The median length of hospitalization was 2 days (IQR: 1; 3). In total, 669 cases (11.0%) were admitted to ICU with 55.3% of ICU cases requiring respiratory support (mechanical ventilation: 31.4%; noninvasive respiratory support: 23.9%). Antiviral use was uncommon with only 1302 cases (21.5%) receiving antiviral therapy. Of the 25 children with test‐positive influenza who died in hospital, 13 (52.0%) were male, 3 (12.0%) identified as Aboriginal, and 6 (24.0%) were younger than 6 months at time of hospital admission.

### 3.2 The prevalence and impact of comorbidities

In total, 2469 cases (40.8%) were reported to have at least one comorbidity with chronic respiratory comorbidities most prevalent (13.8%; Table 1). Of children with comorbidities, 37.5% (*n* = 926) reported more than one comorbidity. Influenza‐confirmed cases with comorbidities were older, more likely to be vaccinated for influenza, and have nosocomial influenza compared with their otherwise healthy peers with influenza (Table 2). Those with comorbidities were more likely to experience severe influenza disease with higher rates of ICU admission (15.7% vs. 7.9%; *p* < 0.001), longer hospitalization (3 versus 1 day; *p* < 0.001), and higher in‐hospital, all‐cause mortality (0.7% vs. 0.3%; *p* = 0.02; Table 2). In addition, interventions were greater in those with comorbidities, including mechanical ventilation (5.0% vs. 2.4%; *p* < 0.001), noninvasive respiratory support (4.7% vs. 1.3%; *p* < 0.001), and antiviral use (33.7% vs. 13.1%; *p* < 0.001) (Table 2). Differences in demographics and clinical outcomes by comorbidity type are summarized in supporting information Table S2.

**TABLE 2:** Characteristics of hospitalized influenza‐confirmed cases with and without comorbidities

Variable	Comorbidity status, no. (%)			Chi‐square^a^ or Mann–Whitney *U* ^b^ test scores (*χ* ^2^ and *z*) and *p*‐values
	Children without comorbidities (*n* = 3588)	Children with comorbidities (*n* = 2469)	All hospitalized influenza‐confirmed children (*n* = 6057)	
**Demographics**				
Male	2004 (56.0%)	1368 (55.4%)	3372 (55.7%)	*χ* ^2^ = 0.2; *p* = 0.68
Aboriginal	297 (8.4%)	184 (7.6%)	481 (8.1%)	*χ* ^2^ = 1. 5; *p* = 0.23
Median age at admission, years (IQR)	2.9 (1.0; 6.3)	4.7 (1.8; 8.5)	3.6 (1.2; 7.2)	z = −13.1*;* ***p* < 0.001**
**Clinical factors**				
Current influenza vaccination	221 (6.2%)	385 (15.6%)	606 (10.0%)	*χ* ^2^ = 146.3; ***p* < 0.001**
Nosocomial infection	142 (4.0%)	253 (10.3%)	395 (6.5%)	*χ* ^2^ = 0.2; ***p* < 0.001**
**Treatments**				
Antiviral use	469 (13.1%)	833 (33.7)	1302 (21.5%)	*χ* ^2^ = 0.2*;* ***p* < 0.001**
Oxygen support	177 (4.9%)	132 (5.4%)	309 (5.1%)	*χ* ^2^ = 0.5; *p* = 0.47
Noninvasive support (CPAP/BiPAP)	46 (1.3%)	116 (4.7%)	162 (2.7%)	*χ* ^2^ = 65.5; ***p* < 0.001**
Mechanical ventilation	86 (2.4%)	124 (5.0%)	210 (3.5%)	*χ* ^2^ = 30.1; ***p* < 0.001**
ECMO	9 (0.3%)	5 (0.2%)	14 (0.2%)	*χ* ^2^ = 0.2; *p* = 0.70
**Hospitalization outcomes**				
Length of hospitalisation; median, days (IQR)	1 (1; 3	3 (1; 5)	2 (1; 3)	z = −20.1; ***p* < 0.001**
ICU admission	282 (7.9%)	387 (15.7%)	669 (11.1%)	*χ* ^2^ = 90.9: ***p* < 0.001**
ICU LOS: median, days (IQR)	2 (1; 4)	3 (1; 7)	2 (1; 6)	z = −3.2; ***p* = 0.002**
Mortality	9 (0.3%)	16 (0.7%)	25 (0.4%)	*χ* ^2^ = 5.6*;* ***p* = 0.02**

*Note*: Values in bold are statistically significant as is standard.

Abbreviations: BiPAP, Bilevel Positive Airway Pressure; CPAP, Continuous positive airway pressure; ECMO, extracorporeal membrane oxygenation; ICU, intensive care unit.

^a^ Chi‐square testing was used to evaluate significance differences between variables in children with and without comorbidities except for age, length of hospitalization and length of ICU stay.

^b^ Mann–Whitney *U* tests were used to evaluate differences in age, length of hospitalization, and length of ICU stay.

Comorbidities were an independent predictor of severe outcomes. Specifically, the odds of ICU admission were higher in those with any comorbidity (aOR: 1.36, 95% Confidence Interval [95% CI]: 1.05; 1.77) compared with cases without any comorbidity. Children with diabetes (aOR: 3.22, 95% CI: 1.25; 8.23), cardiac (aOR: 1.93, 95% CI: 1.23; 3.03), respiratory (aOR: 1.54, 95% CI: 1.08; 2.21), or neurological comorbidities (aOR: 1.57, 95% CI: 1.25; 1.98) were at greatest odds of ICU admission (Table 3). Of note, children with immunosuppression and/or malignancy were at lower odds of ICU admission (aOR: 0.28, 95% CI: 0.17; 0.45). Respiratory, neurological, cardiac, genetic, and hepatic comorbidities and diabetes were associated with prolonged hospitalization, whereas respiratory, renal, cardiac, and other comorbidities increased the length of ICU stay (supporting information Table S3). All‐cause mortality was more likely in cases with neurological comorbidities (aOR: 3.31, 95% CI: 1.79; 6.13), immunosuppression and/or malignancies (aOR: 1.85, 95% CI: 1.10; 3.14), and genetic comorbidities (aOR: 1.97, 95% CI: 1.38; 2.83, supporting information Table S4).

**TABLE 3:** Factors associated with ICU admission and mechanical ventilation in influenza‐positive children

Variable (*N* = patient no. with variable)	ICU admission (*n* = 549)			Mechanical ventilation (*n* = 210)		
	*n* (% of children with variable and ICU admission)	Crude odds ratio (95% CI)	Adjusted odds ratio (95% CI)	*n* (% of children with variable and mechanical ventilation)	Crude odds ratio (95% CI)	Adjusted odds ratio (95% CI)
**Comorbidities**						
No comorbidities (*N* = 3588)	282 (7.9%)	Reference	Reference	86 (2.4%)	Reference	Reference
Any comorbidity (*N* = 2469)	387 (15.7%)	2.18 (1.85; 2.56)	**1.36 (1.05; 1.75)**	124 (5.0%)	2.15 (1.63; 2.85)	1.08 (0.68; 1.71)
Cardiac comorbidity (*N* = 330)	87 (26.4%)	3.17 (2.44; 4.10)	**1.93 (1.23; 3.03)**	40 (12.1%)	4.51 (3.13; 6.49)	**2.46 (1.45; 4.17)**
Diabetic comorbidity (*N* = 76)	22 (29.0%)	3.36 (2.03; 5.55)	**3.22 (1.25; 8.23)**	2 (2.6%)	0.75 (0.18; 3.08)	0.68 (0.26; 1.77)
Genetic comorbidity (*N* = 271)	42 (15.5%)	1.51 (1.07; 2.12)	0.80 (0.60; 1.06)	15 (5.5%)	1.68 (0.98; 2.88)	0.79 (0.58; 1.09)
Hepatic comorbidity (*N* = 122)	17 (13.9%)	1.31 (0.78; 2.02)	1.01 (0.49; 2.11)	8 (6.6%)	1.99 (0.96; 4.13)	**2.32 (1.12; 4.80)**
Immunosuppression and/or malignancies (*N* = 614)	48 (7.8%)	0.66 (0.48; 0.89)	**0.28 (0.17; 0.45)**	15 (2.4%)	0.67 (0.40; 1.15)	**0.32 (0.14; 0.75)**
Neurological comorbidity (*N* = 620)	132 (21.3%)	2.47 (2.00; 3.05)	**1.57 (1.25; 1.98)**	1 (3.0%)	0.87 (0.12; 6.39)	**1.93 (1.12; 2.64)**
Obesity (N = 37)	5 (13.5%)	1.26 (0.49; 3.25)	0.81 (0.30; 2.21)	50 (8.1%)	2.89 (2.08; 4.02)	0.51 (0.13; 2.05)
Other comorbidities (*N* = 33)	4 (12.1%)	1.11 (0.39; 3.17)	0.67 (0.21; 2.11)	2 (5.4%)	1.60 (0.38; 6.68)	0.76 (0.09; 1.59)
Renal comorbidity (*N* = 173)	22 (12.7%)	1.18 (0.75; 1.86)	0.72 (0.40; 1.32)	7 (4.1%)	1.18 (0.55; 2.55)	0.83 (0.24; 2.83)
Respiratory comorbidity (*N* = 834)	171 (20.5%)	2.45 (2.02; 2.96)	**1.54 (1.08; 2.21)**	51 (6.1%)	2.07 (1.50; 2.87)	1.17 (0.61; 2.26)
**Influenza type**						
Influenza A (*N* = 4162)	479 (11.5%)	Reference	Reference	156 (3.8%)	Reference	Reference
Influenza B (*N* = 1855)	186 (10.0%)	0.86 (0.72; 1.02)	1.01 (0.90; 1.14)	52 (2.8%)	0.74 (0.54; 1.02)	0.82 (0.53; 1.25)
Multiple influenza strains (*N* = 40)	4 (10.0%)	0.85 (0.30; 2.41)	**0.60 (0.36; 0.99)**	2 (5.0%)	1.35 (0.32; 5.65)	0.60 (0.33; 1.10)
**Clinical factors**						
Nosocomial infection	116 (29.4%)	3.84 (3.04; 4.85)	**3.26 (1.71; 6.22)**	56 (14.2%)	5.91 (4.27; 8.18)	**5.20 (2.87; 9.43)**
Antiviral use	304 (23.4%)	3.66 (3.10; 4.33)	**4.59 (2.81; 7.51)**	122 (9.4%)	5.48 (4.14; 7.27)	**5.80 (4.07; 8.25)**
Current influenza vaccination	74 (10.3%)	0.91 (0.70; 1.17)	0.74 (0.52; 1.05)	20 (2.8%)	0.73 (0.46; 1.17)	**0.61 (0.37; 0.99)**
**Age at admission**						
≥5 years (*N* = 2394)	236 (9.8%)	Reference	Reference	68 (2.8%)	Reference	Reference
24–59 months (*N* = 1572)	149 (9.5%)	0.80 (0.59; 1.10)	1.17 (0.96; 1.42)	46 (2.9%)	0.69 (0.41; 1.18)	1.20 (0.81; 1.76)
12–23 months (*N* = 797)	87 (10.9%)	0.63 (0.47; 0.84)	**1.51 (1.18; 1.92)**	33 (4.1%)	0.74 (0.46; 1.17)	**1.78 (1.46; 2.17)**
6–11 months (*N* = 537)	73 (13.6%)	0.53 (0.41; 0.69)	**2.29 (1.84; 2.86)**	21 (3.9%)	0.51 (0.33; 0.79)	**1.89 (1.03; 3.45)**
<6 months (*N* = 757)	124 (16.4%)	0.56 (0.44; 0.71)	**1.94 (1.67; 2.26)**	42 (5.6%)	0.50 (0.34; 0.74)	1.29 (0.81; 1.76)
≥5 years (*N* = 2394)	236 (9.8%)	Reference	Reference	68 (2.8%)	Reference	Reference
**Demographics**						
Female sex (*N* = 2685)	289 (10.8%)	Reference	Reference	99 (3.7%)	Reference	Reference
Male sex (*N* = 3372)	379 (11.2%)	1.05 (0.89; 1.23)	1.10 (0.97; 1.25)	111 (3.3%)	0.89 (0.67; 1.17)	0.86 (0.64; 1.16)
Non‐Aboriginal (*N* = 5576)	607 (11.1%)	Reference	Reference	190 (3.5%)	Reference	Reference
Aboriginal (*N* = 481)	54 (11.2%)	1.01 (0.76; 1.36)	1.01 (0.48; 2.12)	20 (4.2%)	1.21 (0.75; 1.93)	1.35 (0.75; 2.44)

*Note*: The logistic regression model was adjusted to sex, age group, Aboriginal and/or Torres Strait Islander status, comorbidities, year of admission, and clustered by Australian state. Values in bold are statistically significant as is standard.

### 3.3 Vaccine coverage and effectiveness

**FIGURE 1:**
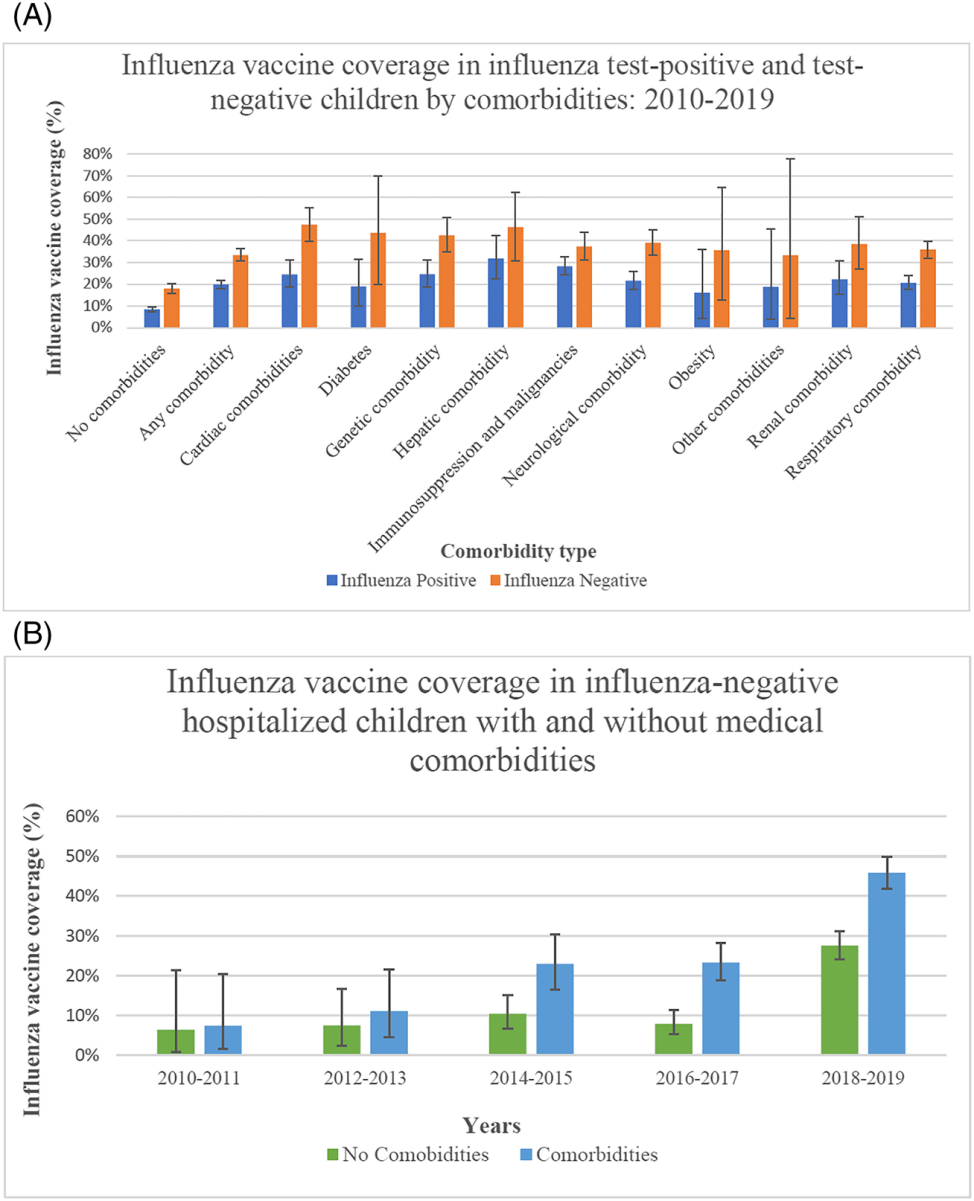
(A) Influenza vaccine coverage in influenza test‐positive and test‐negative children by comorbidities:2010–2019. (B) Influenza vaccine coverage in influenza‐negative hospitalized children with and without comorbidities

For vaccine coverage and VE estimates, 4262 influenza cases and 2488 eligible influenza test‐negative controls were included (supporting information Figure S1). Vaccine coverage in those with and without influenza was 13.2% (95% CI: 12.2%; 14.2%) and 25.4% (95% CI: 23.7%; 27.2%), respectively. Overall coverage in influenza test‐negative controls with and without comorbidities was 33.5% (95% CI: 30.8%; 36.3%) and 17.9% (95% CI: 15.8%; 20.1%), respectively. Coverage ranged from 47.5% (95% CI: 40.0%; 55.1%) in influenza test‐negative children with cardiac comorbidities to 33.3% (95% CI: 4.3%; 77.7%) in influenza‐negative children with other comorbidities (long‐term aspirin therapy and inborn errors of metabolism, Figure 1A). With funded vaccination for all children aged 6 months to <5 years introduced almost‐universally in 2018, overall vaccine coverage rose between 2016–2017 and 2018–2019 from 23.4% (95% CI: 23.7%; 27.2%) to 45.9% (95% CI: 41.8%; 49.9%) in influenza test‐negative children with comorbidities and 8.0% (95% CI: 5.3%; 11.4%) to 27.6% (95% CI: 24.2%; 31.3%) in influenza test‐negative children without comorbidities (Figure 1B). Similar increases were observed for those aged >5 years old with comorbidities (from 32.1% [95% CI: 23.6%; 41.6%] to 47.0% [95% CI: 39.5%; 54.6%] and without comorbidities (from 2.3% [95% CI: 0.1%; 12.0%] to 20.5% [95% CI: 12.4%; 30.8%]).

Overall, VE against hospitalized influenza was estimated to be 57% (95% CI: 50%; 63%). Significant VE ranged from 76% (95% CI: 60%; 86%) for Influenza A/H1N1 to 43% (95% CI: 19%; 60%) for A/H3N2. Influenza B had comparable VE to the overall VE at 56% (95% CI: 44%; 65%). Of note, a statistically significant VE was demonstrated in all age groups 12 months (Table 4) and older and for Aboriginal children (51%, 95% CI: 2%; 76%). Similar VE was demonstrated in those with (55%, 95% CI: 45%; 63%) and without (57%, 95% CI: 44%; 67%) comorbidities. The VE for those with respiratory comorbidities was 64% (95% CI: 49%; 74%), 75% (95% CI: 50%; 87%) for cardiac, 64% (95% CI: 44%; 77%) for neurological, and 62% (95% CI: 29%; 80%) for genetic comorbidities (Table 4). We did not show significant VE against influenza‐associated hospitalization in children with immunosuppression and/or malignancies (23%, 95% CI: −22%; 51%).

**TABLE 4:** Influenza vaccine effectiveness in hospitalized Australian children by influenza strains, comorbidities, and age groups for all years combined (2010–2019)

Variable	Positive cases		Controls		VE (95% CI), %
	Vaccinated (*n* = 562)	Unvaccinated (*n* = 3700)	Vaccinated (*n* = 632)	Unvaccinated (*n* = 1856)	Adjusted VE^a^
**Influenza strains**					
All strains	562	3700	632	1856	**57% (50%; 63%)**
Influenza A	364	2549	632	1856	**57% (49%; 64%)**
H1N1	22	402	632	1856	**76% (60%; 86%)**
H3N2	65	403	632	1856	**43% (19%; 60%)**
H untyped	277	1744	632	1856	**55% (45%; 63%)**
Influenza B	198	1151	632	1856	**56% (44%; 65%)**
**Age at admission**					
6–11 months	64	375	99	406	26% (−17%; 53%)
12–23 months	87	576	186	531	**53% (34%; 67%)**
24–59 months	171	1104	196	494	**61% (49%; 71%)**
≥5 years	240	1645	151	425	**60% (47%; 70%)**
**Demographics**					
Aboriginal	48	267	39	112	**51% (2%; 76%)**
Male sex	303	2049	376	1065^b^	**60% (51%; 68%)**
Female sex	259	1651	256	790	**55% (44%; 65%)**
**Comorbidities**					
No comorbidity	213	2293	231	1060	**57% (44%; 67%)**
Any comorbidity	349	1407	401	796	**55% (45%; 63%)**
Cardiac comorbidity	48	148	84	93	**75% (50%; 87%)**
Diabetes	11	47	7	9	‐
Genetic comorbidity	51	157	69	93	**62% (29%; 80%)**
Hepatic comorbidity	28	60	19	22	2% (−207%; 61%)
Immunosuppressed and/or malignancy	126	320	85	142	23% (−22%; 51%)
Neurological comorbidity	93	340	111	173	**64% (44%; 77%)**
Obesity	4	21	5	9	‐
Other comorbidities	3	13	2	4	‐
Renal comorbidity	27	94	27	43	61% (−3%; 85%)
Respiratory comorbidity	127	491	211	376	**64% (49%; 74%)**

*Note*: Values in bold are statistically significant as is standard.

^a^ The conditional logistic regression model was adjusted to sex, age group, Aboriginal and/or Torres Strait Islander status, comorbidities and group matched by Australian state of admission, month of admission and year of admission. Influenza vaccination status was defined as having ≥1 influenza vaccine doses within the calendar year of admission.

^b^ The sex status of one unvaccinated influenza‐negative control was missing.

## 4 DISCUSSION

This is the largest evaluation of pediatric influenza hospitalizations in Australia to date with previous evaluations frequently restricted to single influenza seasons.^6^, ^10^, ^14^ Our results highlight the significant increase in risk of severe influenza in children with medical comorbidities. This was in addition to more severe outcomes in younger children and those with nosocomial influenza. The increased clinical burden, proportion with severe outcomes, and effectiveness of vaccination in children with comorbidities remind us of the importance of targeted vaccination strategies and programs in this vulnerable group.

Comorbidities were associated with increased severity of clinical outcomes including greater odds of ICU admission, extended hospitalization, and in‐hospital mortality. ICU admission and length of hospitalization were greatest in cases with respiratory and/or neurological comorbidities, whereas increased odds of mechanical ventilation were seen for those with cardiac, hepatic, and neurological comorbidities. The association between specific comorbidities and severe outcomes has been observed previously including in a large cohort of 10 173 influenza‐positive children hospitalized in the United States across three influenza seasons.^15^ Cardiac, hepatic, and neurological conditions were significantly associated with mechanical ventilation, consistent with our findings.^16^

We observed that children with immunosuppression and/or malignancies were at lower odds of ICU admission and mechanical ventilation than children without immunosuppression and/or malignancies. Pre‐emptive hospitalization, more aggressive management of children with fever, and greater proactive influenza management in this patient group may account for these differences.^17^

As observed previously,^7^, ^10^ antiviral use was low in Australian children relative to other similar high‐income settings.^18^ Currently, national guidelines recommends that any child hospitalized with confirmed influenza should be given antiviral treatment as well as outpatients such as those with comorbidities at risk of severe outcomes.^16^ Further research on the modifiable factors associated with ongoing low use of antivirals in Australian pediatric hospitals is urgently required. It should be noted that antiviral use was associated with ICU admission, mechanical ventilation, extended hospitalization length, and increased ICU stay, likely due to residual confounding with increased antiviral prescriptions in those with very severe influenza infections.

Children with comorbidities were significantly more likely to have nosocomial influenza than children without comorbidities. Nosocomial infections were independently associated with severe influenza outcomes. Pediatric nosocomial respiratory viral infections have previously been shown to lead to more severe clinical outcomes including mortality and extended hospitalization length.^19^ Further efforts to reduce nosocomial acquisition from family and staff are required for this vulnerable cohort.

Aboriginal children were overrepresented in the influenza‐positive cohort (7.9% of this cohort compared with 3.3% of the general Australian population)^20^ and experience longer hospitalization stays than non‐Aboriginal children. This higher rate of influenza hospitalizations has been previously recognized and precipitated national influenza vaccination funding for all Aboriginal persons aged 6 months and older.^12^ Extended length of hospitalization has additionally been observed in Aboriginal children with other respiratory viral infections.^21^ This is the first test‐negative evaluation to specifically demonstrate VE in Australian Aboriginal children, providing additional evidence to support the current program providing immunization to all Aboriginal children ≥6 months of age.

Influenza vaccination provided comparable protection for children with and without comorbidities against influenza hospitalization. This result is critical given concerns about influenza vaccine responses in those with comorbidities.^22^ This finding highlights the potential impact of higher influenza vaccine coverage; preventing severe influenza outcomes in children already impacted by medical comorbidities. Coverage rates improved over time for all children with consistently higher coverage observed in children with comorbidities. The greatest increase was seen in 2018–2019 postintroduction of a funded influenza vaccine program for all children aged 6 months to <5 years. While a large improvement was observed for children across all ages,^23^ coverage remains suboptimal and well below that of noninfluenza vaccines on the NIP.^24^ Maximizing coverage with current influenza vaccines requires interventions targeting parental attitudes, clinician behaviors, and immunization delivery practices.^25^ It is anticipated that future influenza vaccines currently in development should produce greater protection in children and adults.^26^

Children aged <6 months were at greater odds of being admitted to ICU and experience longer hospital stays. Maternal influenza vaccination provides protection in children's first 6 months of life^27^ and has been publicly funded under the NIP since 2010.^8^ Potential administration of influenza vaccines earlier in infancy (i.e. <6 months of age) may further reduce disease burden in this population.^28^ Clinical trials are currently underway to assess the safety and immunogenicity of an influenza vaccine given earlier in infancy.^29^ In this analysis, children 6 to 11 months were shown to have a lower VE compared with older children. Although there is some evidence that younger children producing a poorer immune response to inactive influenza vaccines compared with older children,^30^ this has not been a consistent finding.^31^

Our evaluation had several limitations. Firstly, given the nature of sentinel hospital surveillance, we have a limited ability to assess population‐based influenza‐associated hospitalization rates. Despite this, given recruitment was Australia‐wide; we believe that the findings from this study are generalizable to the broader Australian population. The PAEDS‐FluCAN network only captures influenza hospitalizations during the southern hemisphere influenza season (April–October), missing interseasonal cases.^32^ Recruitment of noninfluenza controls was capped during peak influenza seasons to ensure complete case capture, resulting in discrepancy between the number of cases and controls. Typing of influenza viruses was not uniformly performed thereby reducing our capacity to track VE by influenza type/subtype. Patients with a single influenza vaccine dose record in the year of their hospitalization were identified as fully vaccinated. However, due to children 6 months to 9 years old receiving their first influenza immunization in Australia requiring two vaccination doses, this may have caused the level of vaccination to be overestimated.

In summary, influenza‐positive children with medical comorbidities captured from 10 years of hospital surveillance across Australia were more likely to experience longer hospital stay, ICU admission, receive antiviral therapy, and die during hospitalization. Certain comorbidities including cardiac and neurological comorbidities further increased a child's risks for severe influenza disease and worsening outcomes of ICU admission and mechanical ventilation. Influenza vaccination was shown to provide similar protection for children with comorbidities compared with otherwise healthy children. Influenza vaccine coverage has improved in children with and without comorbidities with increased vaccine funding, however, coverage remains inadequate. The PAEDS‐FluCAN collaboration has also captured important data detailing the factors influencing coverage and outcomes of severe influenza disease in children. These results will be used to inform future public health interventions targeting influenza vaccine use and reducing pediatric influenza‐associated morbidity and mortality.

## FUNDING INFORMATION

This work was supported by funding from the National Health and Medical Research Council (NHMRC Partnership Grant: APP1113851), Australian Government Departments of Health and Departments of Health in NSW, Victoria, Queensland, South Australia, Western Australia, and the Northern Territory. FluCAN receives support from the Australian Government Department of Health. A/Prof Blyth, Prof Marshall, and Prof Cheng are supported by NHMRC Fellowships. A/Prof Danchin is Melbourne University's David Bickart Clinician Scientist fellowship recipient.

## AUTHOR CONTRIBUTIONS

**Daniel Norman:** Conceptualization; data curation; formal analysis; investigation; methodology. **Kristine Macartney:** Data curation; formal analysis; funding acquisition; investigation. **Hannah Moore:** Formal analysis; investigation; methodology; supervision; validation. **Holly Seale:** Conceptualization; investigation; supervision. **Margie Danchin:** Conceptualization; data curation; methodology. **Nigel Crawford:** Data curation; formal analysis. **Allen Cheng:** Conceptualization; data curation; formal analysis; funding acquisition; methodology; resources. **Jocelynne McRae:** Data curation; formal analysis. **Jim Buttery:** Conceptualization; methodology. **Helen Marshall:** Data curation; formal analysis. **Julia Clark:** Data curation; formal analysis. **Joshua Francis:** Data curation; formal analysis. **Christopher Blyth:** Conceptualization; formal analysis; investigation; methodology; project administration; supervision.

## Supporting information


Supplementary Table S1
**Supplementary Table S2:** Influenza positive patient characteristics by comorbidity type
**Supplementary Table S3–1:** Influenza positive patient characteristics by comorbidity type
**Supplementary Table S3:** Factors associated with increased length of hospitalisation and ICU stay in Australia's influenza positive children (2010 to 2019)
**Supplementary Table S3–2:** Factors associated with increased length of hospitalisation and ICU stay in Australia's influenza positive children (2010 to 2019)
**Supplementary Table S4:** Factors associated with mortality in Australia's influenza positive children
a: The logistic regression model was adjusted to sex, age group, Aboriginal and/or Torres Strait Islander status and comorbidities with clustering by Australian state

**Supplementary Figure 1:** Flowchart of Children in the epidemiological and vaccine effectiveness (VE) cohort
*The number of influenza positive cases and influenza negative controls are less than the sum of each exclusion criteria due to certain cases and controls having multiple exclusion criteria.

## Data Availability

The data that support the findings of this study are available from the corresponding author upon reasonable request.
