# Supplementary material for: Influenza hospitalizations in Australian children 2010–2019: The impact of medical comorbidities on outcomes, vaccine coverage, and effectiveness
Source: Influenza Other Respir Viruses. 2021 Nov 16;16(2):316–27. doi: 10.1111/irv.12939 (PMC8818821; doi:10.1111/irv.12939)
Supplement: Supplementary file 1 — Supplementary Table S1 Supplementary Table S2: Influenza positive patient characteristics by comorbidity type Supplementary Table S3–1: Influenza positive patient characteristics by comorbidity type Supplementary Table S3: Factors associated with increased length of hospitalisation and ICU stay in Australia's influenza positive children (2010 to 2019) Supplementary Table S3–2: Factors associated with increased length of hospitalisation and ICU stay in Australia's influenza positive children (2010 to 2019) Supplementary Table S4: Factors associated with mortality in Australia's influenza positive children a: The logistic regression model was adjusted to sex, age group, Aboriginal and/or Torres Strait Islander status and comorbidities with clustering by Australian state [file IRV-16-316-s002.docx]

**Supplementary Table 1**

| **Hospital Sites** | **Hospital Type** | **Australian State** | **PAEDS-FluCAN years** | **Influenza cases** |
| --- | --- | --- | --- | --- |
| Children’s Hospital at Westmead | Tertiary Children’s Hospital | New South Wales | 2011-2019 | 1813 |
| John Hunter Hospital | General Hospital | New South Wales | 2010 | 3 |
| Westmead Hospital | General Hospital | New South Wales | 2017-2019 | 2 |
| Royal Children’s Hospital Melbourne | Tertiary Children’s Hospital | Victoria | 2018-2019 | 447 |
| Monash Children’s Hospital | Tertiary Children’s Hospital | Victoria | 2017-2019 | 459 |
| Monash Medical Centre | General Hospital | Victoria | 2010-2014 | 50 |
| University Hospital Geelong | General Hospital | Victoria | 2012-2019 | 221 |
| The Alfred Hospital | General Hospital | Victoria | 2011-2012 | 5 |
| Queensland Children’s Hospital | Tertiary Children’s Hospital | Queensland | 2017-2019 | 603 |
| Cairns Base Hospital | General Hospital | Queensland | 2010-2019 | 205 |
| Mater Hospital | General Hospital | Queensland | 2010-2011 | 3 |
| Princess Alexandra Hospital | General Hospital | Queensland | 2014 | 1 |
| Princess Margaret Hospital / Perth Children’s Hospital | Tertiary Children’s Hospital | Western Australia | 2010-2019 | 1029 |
| Royal Perth Hospital | General Hospital | Western Australia | 2011-2018 | 6 |
| Women’s and Children’s Hospital | Tertiary Children’s Hospital | South Australia | 2011-2013 | 506 |
| Royal Adelaide Hospital | General Hospital | South Australia | 2012-2015 | 7 |
| Royal Hobart Hospital | General Hospital | Tasmania | 2010-2019 | 87 |
| Canberra Hospital | General Hospital | Australian Capital Territory | 2010-2019 | 383 |
| Alice Springs Hospital | General Hospital | Northern Territory | 2010-2019 | 162 |
| Royal Darwin Hospital | General Hospital | Northern Territory | 2017-2019 | 65 |

| **Variable**  **Supplementary Table 2:** Influenza positive patient characteristics by comorbidity type  **Supplementary Table 3-1:** Influenza positive patient characteristics by comorbidity type | **Comorbidity type (Patient, No. [% of total population])** | | | | | | | | | |
| --- | --- | --- | --- | --- | --- | --- | --- | --- | --- | --- |
|  | Respiratory comorbidity  (n = 834, 13.8%) | Neurological comorbidity  (n = 620, 10.2%) | Immunosuppression and/or malignancies  (n = 614,  10.1%) | Cardiac comorbidity  (n = 330, 5.4%) | Genetic comorbidity  (n = 271, 4.5%) | Renal comorbidity  (n = 173, 2.9%) | Hepatic comorbidity  (n = 122, 2.0%) | Diabetes  (n = 76, 1.3%) | Obesity  (n = 37, 0.6%) | Other comorbidities  (n = 33,  0.5%) |
| **Demographic** | | | | | | | | | | |
| Male | 475 (57.0%) | 339 (54.7%) | 341 (55.5%) | 169 (51.2%) | 139 (51.3%) | 90 (52.0%) | 58 (47.5%) | 38 (50.0%) | 21 (56.8%) | 19 (57.6%) |
| Aboriginal | 82 (9.8%) | 46 (7.4%) | 31 (5.1%) | 28 (8.5%) | 21 (7.8%) | 7 (4.1%) | 4 (3.3%) | 2 (2.6%) | 5 (13.5%) | 0 (0.0%) |
| Median age at admission, years (IQR) | 5.5  (2.1; 9.4) | 5.4  (2.5; 9.6) | 5.8  (3.5; 9.7) | 2.7  (0.6; 6.5) | 5.6  (2.6; 10.2) | 4.8  (2.0; 8.0) | 5.5  (2.7; 8.7) | 8.3(4.8; 13.4) | 10.7  (5.9; 13.3) | 2.9  (1.1; 4.3) |
| **Clinical factors** | | | | | | | | | | |
| Nosocomial infection | 93 (11.2%) | 79 (12.7%) | 70 (11.4%) | 55 (16.7%) | 30 (11.1%) | 23 (13.3%) | 13 (10.7%) | 11 (14.5%) | 3 (8.1%) | 4 (12.1%) |
| Current influenza vaccination | 146 (17.5%) | 103 (16.1%) | 138 (22.5%) | 57 (19.9%) | 58 (21.4%) | 30 (17.3%) | 31 (25.4%) | 13 (17.1%) | 4 (10.8%) | 3 (9.1%) |
| **Number of additional comorbidity types** | | | | | | | | | | |
| 1 | 250 (30.0%) | 183 (29.5%) | 176 (28.7%) | 176 (27.2%) | 88 (32.5%) | 59 (34.1%) | 55 (45.1%) | 41 (59.0%) | 10 (27.0%) | 8 (24.2%) |
| 2 | 130 (15.6%) | 123 (19.9%) | 55 (9.0%) | 102 (15.8%) | 77 (28.4%) | 45 (26.0%) | 28 (23.0%) | 15 (19.7%) | 6 (16.2%) | 11 (33.3%) |
| 3 | 52 (6.2%) | 45 (7.3%) | 26 (4.2%) | 33 (5.1%) | 34 (12.6%) | 18 (10.4%) | 16 (13.1%) | 11 (14.5%) | 3 (8.1%) | 6 (18.2%) |
| 4 or more | 19 (2.3%) | 20 (3.2%) | 12 (2.0%) | 12 (1.9%) | 16 (5.9%) | 7 (4.1%) | 4 (3.3%) | 9 (11.8%) | 3 (8.1%) | 0 (0.0%) |
| **Treatments** | | | | | | | | | | |
| Antiviral use | 244 (29.3%) | 207 (33.4%) | 379 (61.4%) | 106 (32.1%) | 92 (34.0%) | 57 (33.0%) | 20 (16.4%) | 20 (26.3%) | 16 (43.2%) | 8 (24.2%) |
| Oxygen support | 51 (6.1%) | 29 (4.7%) | 24 (3.9%) | 19 (5.8%) | 6 (2.2%) | 9 (5.2%) | 18 (14.8%) | 18 (23.7%) | 0 (0.0%) | 1 (3.0%) |
| Non-invasive support (CPAP/BiPAP) | 73 (8.8%) | 46 (7.4%) | 5 (0.8%) | 21 (6.4%) | 12 (4.4%) | 5 (2.9%) | 2 (1.6%) | 2 (2.6%) | 2 (5.4%) | 2 (6.1%) |
| Mechanical ventilation | 51 (6.1%) | 50 (8.1%) | 1 (4.8%) | 40 (12.1%) | 15 (5.5%) | 7 (4.1%) | 2 (1.6%) | 2 (2.6%) | 2 (5.4%) | 1 (3.0%) |
| ECMO | 2 (0.2%) | 1 (0.2%) | 0 (0.0%) | 2 (0.6%) | 0 (0.0%) | 0 (0.0%) | 1 (0.8%) | 1 (0.4%) | 0 (0.0%) | 0 (0.0%) |
| **Hospitalisation outcomes** | | | | | | | | | | |
| Hospitalisation length; median days (IQR) | 3 (1; 7) | 3 (2; 8) | 3 (2; 5) | 3 (2; 8) | 3 (1; 7) | 2 (1; 5) | 3 (2; 4) | 4 (2; 6) | 2 (1; 5) | 4 (2; 14) |
| ICU admission | 171 (20.5%) | 132 (21.3%) | 48 (7.8%) | 87 (26.4%) | 42 (15.5%) | 22 (12.7%) | 17 (13.9%) | 22 (29.0%) | 5 (13.5%) | 4 (12.1%) |
| Length of ICU stay; median, days (IQR) | 3 (1; 8) | 3.5 (2; 9) | 4 (2; 8) | 6 (2; 15) | 3 (2; 12) | 2 (2; 7) | 8 (2; 19.5) | 1 (1; 3) | 2 (1.5; 10) | 3* |
| Mortality | 7 (0.9%) | 7 (1.2%) | 5 (0.8%) | 6 (1.9%) | 4 (1.5%) | 2 (1.2%) | 1 (0.8%) | 0 (0.0%) | 0 (0.0%) | 1 (3.0%) |

*****All cases with other comorbidities admitted to ICU had length of ICU stays of 3 days.

| **Variable**  **Supplementary Table 3:** Factors associated with increased length of hospitalisation and ICU stay in Australia’s influenza positive children (2010 to 2019)  **Supplementary Table 3-2:** Factors associated with increased length of hospitalisation and ICU stay in Australia’s influenza positive children (2010 to 2019)  **(N = Patient No. with variable)** | **Length of hospitalisation in influenza positive cases, (n = 6057)** | | **Length of ICU stay in influenza positive cases.**  **(n = 445)** | |
| --- | --- | --- | --- | --- |
|  | **Crude incidence rate ratio**  **(95% CI)** | **Adjusted incidence rate ratio^a^**  **(95% CI)** | **Crude incidence rate ratio**  **(95% CI)** | **Adjusted incidence rate ratio^a^**  **(95% CI)** |
| **Comorbidities** | | | | |
| No comorbidities (N = 3588) | Reference | Reference | Reference | Reference |
| Any comorbidity (N = 2469) | 2.42 (2.27; 2.58) | **1.17 (1.03; 1.33)** | 2.41 (1.90; 3.07) | **0.76 (0.60; 0.96)** |
| Cardiac comorbidity (N = 330) | 2.88 (2.51; 3.32) | **1.43 (1.16; 1.77)** | 3.56 (2.61; 4.86) | **1.50 (1.25; 1.80)** |
| Diabetes (N = 76) | 1.65 (1.23; 2.21) | **1.25 (1.10; 1.42)** | 0.35 (0.18; 0.68) | **0.36 (0.28; 0.45)** |
| Genetic comorbidity (N = 271) | 2.28 (1.96; 2.66) | **1.19 (1.12; 1.27)** | 2.21 (1.37; 3.55) | **0.81 (0.67; 0.99)** |
| Hepatic comorbidity (N = 122) | 2.35 (1.87; 2.95) | **1.78 (1.43; 2.21)** | 1.52 (0.70; 3.31) | 1.67 (0.97; 2.87) |
| Immunosuppression and/or malignancies  (N = 614) | 1.77 (1.59; 1.97) | 0.96 (0.83; 1.09) | 1.31 (0.79; 2.20) | 0.66 (0.43; 1.02) |
| Neurological comorbidity (N = 620) | 2.46 (2.22; 2.73) | **1.43 (1.22; 1.68)** | 2.46 (1.84; 3.27) | 1.28 (0.85; 1.94) |
| Obesity (N = 37) | 1.15 (0.75; 1.76) | 0.83 (0.65; 1.05) | 0.70 (0.18; 2.78) | 0.61 (0.30; 1.26) |
| Other comorbidities (N = 33) | 2.65 (1.73; 4.05) | **1.40 (1.01; 1.92)** | 0.37 (0.02; 6.34) | **5.53 (2.89; 10.58)** |
| Renal comorbidity (N = 173) | 1.27 (1.04; 1.55) | 0.91 (0.64; 1.29) | 3.71 (1.83; 7.50) | **1.68 (1.05; 2.69)** |
| Respiratory comorbidity (N = 834) | 2.01 (1.83; 2.21) | **1.33 (1.16; 1.54)** | 1.69 (1.29; 2.22) | **1.25 (1.02; 1.53)** |
| **Influenza type** | | | | |
| Influenza A (N = 4162) | Reference | Reference | Reference | Reference |
| Influenza B (N = 1855) | 1.04 (0.97; 1.12) | 1.08 (0.99; 1.18) | 0.95 (0.73; 1.25) | 1.17 (0.95; 1.44) |
| Multiple Influenza strains (N = 40) | 0.72 (0.47; 1.10) | 0.75 (0.44; 1.29) | 2.19 (0.58; 8.32) | 2.41 (0.72; 8.00) |
| **Demographics** | | | | |
| Female sex (N = 2685) | Reference | Reference | Reference | Reference |
| Male sex (N = 3372) | 0.94 (0.88; 1.01) | 0.96 (0.90; 1.02) | 0.82 (0.64; 1.05) | 0.85 (0.68; 1.07) |
| Non-Aboriginal (N = 5576) | Reference | Reference | Reference | Reference |
| Aboriginal (N = 481) | 1.43 (1.26; 1.61) | **1.45 (1.34; 1.59)** | 1.04 (0.67; 1.63) | 0.85 (0.60; 1.21) |
| **Clinical factors** | | | | |
| Community-acquired infection (N = 5662) | Reference | Reference | Reference | Reference |
| Nosocomial infection (N = 395) | 8.76 (7.86; 9.77) | **6.39 (5.01; 8.14)** | 6.29 (4.87; 8.13) | **4.88 (3.04; 7.82)** |
| Antiviral use (N = 1302) | 2.29 (2.12; 2.47) | **1.89 (1.68; 2.13)** | 1.52 (1.19; 1.93) | **1.73 (1.59; 1.88)** |
| Current influenza vaccination (N = 606) | 1.16 (1.05; 1.28) | 1.06 (0.92; 1.23) | 1.04 (0.70; 1.54) | 0.96 (0.53; 1.71) |
| **Age at admission** | | | | |
| ≥5 years (N = 2394) | Reference | Reference | Reference | Reference |
| 24 - 59 months (N = 1572) | 0.90 (0.83; 0.98) | 1.01 (0.94; 1.09) | 0.82 (0.60; 1.11) | **0.84 (0.73; 0.97)** |
| 12 - 23 months (N = 797) | 0.80 (0.72; 0.89) | 1.01 (0.90; 1.12) | 0.72 (0.50; 1.04) | **0.71 (0.54; 0.93)** |
| 6 - 11 months (N = 537) | 1.00 (0.89; 1.13) | **1.23 (1.09; 1.39)** | 0.57 (0.38; 0.85) | **0.61 (0.40; 0.96)** |
| <6 months (N = 757) | 1.83 (1.65; 2.03) | **1.20 (1.03; 1.41)** | 3.50 (2.57; 4.76) | 1.16 (0.79; 1.69) |

a: Negative binomial regression models were adjusted to sex, age group, Aboriginal and/or Torres Strait Islander status, comorbidities and clustered by Australian state.

| **Variable**  **(N = Patient No. with variable)** | **Mortality** | | |
| --- | --- | --- | --- |
|  | Mortality No. (n)  (Case fatality rate; n/N) | Crude odds ratio (95% CI) | Adjusted odds ratio^a^ (95% CI) |
| **Comorbidities** | | | |
| No comorbidities (N = 3588) | 9 (0.3%) | Reference | Reference |
| Any comorbidity (N = 2469) | 16 (0.7%) | 2.59 (1.14; 5.87) | 1.01 (0.48; 2.09) |
| Cardiac comorbidity (N = 330) | 6 (1.6%) | 5.73 (2.27; 5.87) | 1.31 (0.41; 4.20) |
| Diabetes (N = 76) | 0 (0%) | - | - |
| Genetic comorbidity (N = 271) | 4 (1.5%) | 4.11 (1.40; 12.06) | **1.97 (1.38; 2.83)** |
| Hepatic comorbidity (N = 122) | 1 (0.8%) | 2.04 (0.27; 15.20) | - |
| Immunosuppression and/or malignancies  (N = 614) | 5 (0.9%) | 2.23 (0.84; 5.97) | **1.85 (1.10; 3.14)** |
| Neurological comorbidity (N = 620) | 7 (1.2%) | 3.44 (1.43; 8.26) | **3.31 (1.79; 6.13)** |
| Obesity (N = 37) | 0 (0%) | - | - |
| Other comorbidities (N = 33) | 1 (4.0%) | 7.63 (1.00; 58.12) | - |
| Renal comorbidity (N = 173) | 2 (1.2%) | 3.00 (0.70; 12.83) | 0.80 (0.17; 3.74) |
| Respiratory comorbidity (N = 834) | 7 (0.9%) | 2.44 (1.01; 5.85) | 2.02 (0.95; 4.31) |
| **Influenza type** | | | |
| Influenza A (N = 4162) | 14 (0.3%) | Reference | Reference |
| Influenza B (N = 1855) | 11 (0.6%) | 1.76 (0.80; 3.89) | **3.66 (2.00; 6.70)** |
| Multiple Influenza strains (N = 40) | 0 (0%) | - | - |
| **Clinical factors** | | | |
| Community-acquired infection (N = 5662) | 16 (0.3%) | Reference | Reference |
| Nosocomial infection (N = 395) | 9 (2.5%) | 8.93 (3.91; 20.36) | **13.53 (5.67; 32.26)** |
| Antiviral use (N = 1302) | 12 (1.0%) | 3.43 (1.56; 7.54) | 2.75 (0.91; 8.35**)** |
| Current influenza vaccination (N = 606) | 2 (0.3%) | 0.51 (0.12; 2.09) | 0.56 (0.19; 2.67) |
| **Age at admission** | | | |
| ≥5 years (N = 2394) | 10 (0.4%) | Reference | Reference |
| 24 - 59 months (N = 1572) | 8 (0.5%) | 1.23 (0.83; 1.81) | **2.43 (1.13; 5.22)** |
| 12 - 23 months (N = 797) | 1 (0.1%) | 0.39 (0.05; 1.92) | 0.79 (0.29; 2.13**)** |
| 6 - 11 months (N = 537) | 0 (0%) | - | **-** |
| <6 months (N = 757) | 6 (0.8%) | 1.94 (0.60; 6.81) | **3.14 (1.55; 6.36)** |
| **Demographics** | | | |
| Female sex (N = 2685) | 12 (0.5%) | Reference | Reference |
| Male sex (N = 3372) | 13 (0.4%) | 0.87 (0.39; 1.90) | **0.66 (0.47; 0.92)** |
| Non-Aboriginal (N = 5576) | 22 (0.4%) | Reference | Reference |
| Aboriginal (N = 481) | 3 (0.7%) | 1.63 (0.49; 5.47) | 2.53 (0.66; 9.66) |

**Supplementary Table 4:** Factors associated with mortality in Australia’s influenza positive children

a: The logistic regression model was adjusted to sex, age group, Aboriginal and/or Torres Strait Islander status and comorbidities with clustering by Australian state
