# Supplementary figures and images for: Influenza hospitalizations in Australian children 2010–2019: The impact of medical comorbidities on outcomes, vaccine coverage, and effectiveness
Source: Influenza Other Respir Viruses. 2021 Nov 16;16(2):316–27. doi: 10.1111/irv.12939 (PMC8818821; doi:10.1111/irv.12939)

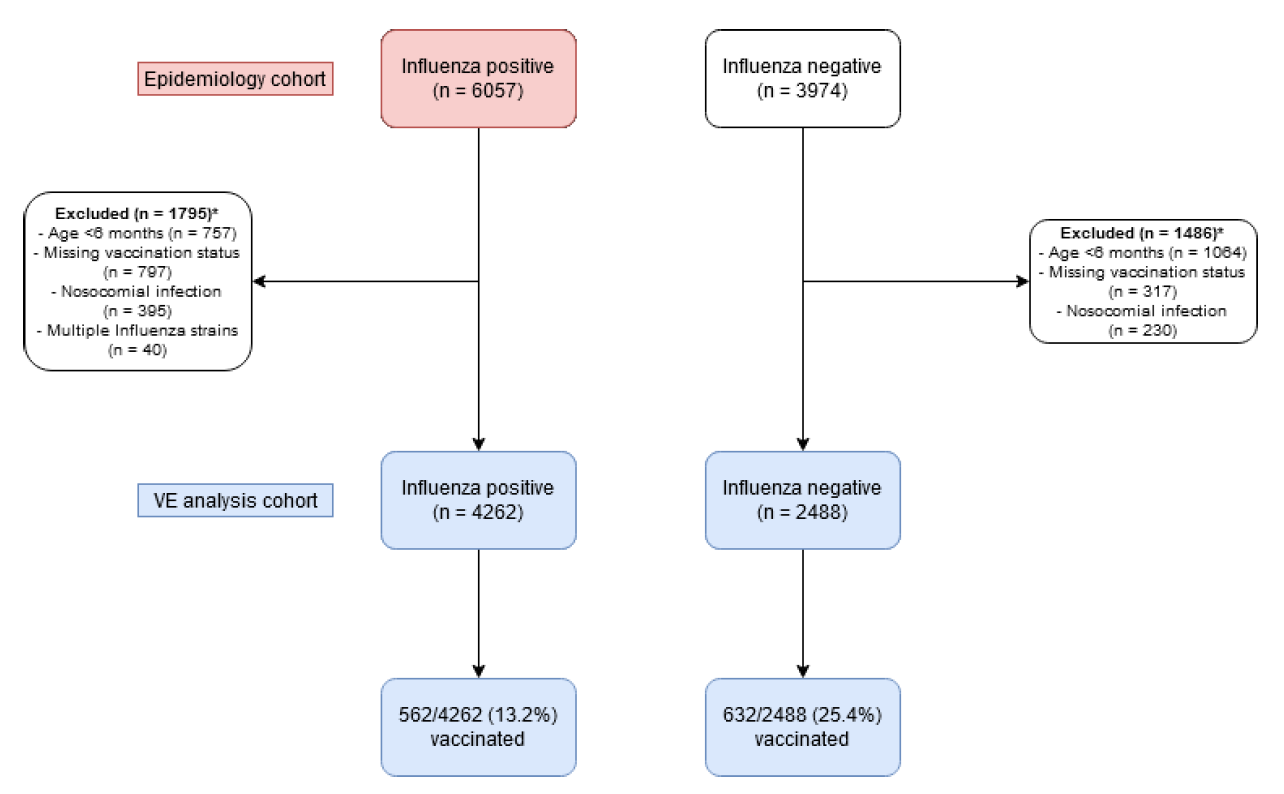

Supplement: Supplementary file 2 — Supplementary Figure 1: Flowchart of Children in the epidemiological and vaccine effectiveness (VE) cohort *The number of influenza positive cases and influenza negative controls are less than the sum of each exclusion criteria due to certain cases and controls having multiple exclusion criteria. [file IRV-16-316-s001.PNG]
